# A Manual of Chemistry

**Published:** 1909-12

**Authors:** 


**A Manual of Chemistry.** A textbook specially adapted for students of medicine, pharmacy and dentistry. By **W. Simon**, Ph.D., M.D., Professor of Chemistry in the College of Physicians and Surgeons, Baltimore, and in the Baltimore College of Dental Surgery, and **Daniel Base**, Ph.D., Professor of Chemistry in the Maryland College of Pharmacy. Ninth edition. Octavo, 716 pages, with 78 engravings and 9 colored plates. Lea & Febiger, Philadelphia and New York. 1909. (Cloth, $3.00, net.)

In this new edition of his manual, increased in size to the extent of 73 pages, Simon has preserved the plan and characteristics that has won for it a marked degree of approval as shown in the exhaustion of the eight previous editions. The book has been thoroughly revised, and brought up to date in all its parts, having been practically rewritten by the author and his colleague Professor Base. While it is stated in the preface that the manual is written for students of medicine, pharmacy and dentistry, an examination of its contents would indicate that a mastery of the book will give the physician a knowledge of chemistry commensurate with his needs.

Among the changes worthy of mention is an extension of the article on crystals, the rearrangement of the article on heat, an increase in the number of experiments and tests, a reconstruction of the chapter dealing with proteins, and the introduction of many new compounds of medical interest. It is believed that the author has well attained his object—namely, "To furnish to the student in concise form a clear presentation of the science, an intelligent discussion of those substances which are of interest to him, and a trustworthy guide to his work in the laboratory." J. A. R.

